# Genetic Predisposition and its Heredity in the Context of Increased Prevalence of Dermatophytoses

**DOI:** 10.1007/s11046-021-00529-1

**Published:** 2021-02-01

**Authors:** Sebastian Gnat, Dominik Łagowski, Aneta Nowakiewicz

**Affiliations:** Faculty of Veterinary Medicine, Institute of Preclinical Veterinary Sciences, Department of Veterinary Microbiology, University of Life Sciences, Akademicka 12, 20-033 Lublin, Poland

**Keywords:** Dermatophytosis, Genetic proneness, Heredity, Predisposing factors, Prevalence, Susceptibility, Immunology, Pathogenicity, Transmission, Etiology

## Abstract

Dermatophytosis is a widespread disease with high prevalence and a substantial economic burden associated with costs of treatment. The pattern of this infectious disease covers a wide spectrum from exposed individuals without symptoms to those with acutely inflammatory or non-inflammatory, chronic to invasive, and life-threatening symptoms. Moreover, the prevalence of cutaneous fungal infections is not as high as might be expected. This curious disparity in the dermatophyte infection patterns may suggest that there are individual factors that predispose to infection, with genetics as an increasingly well-known determinant. In this review, we describe recent findings about the genetic predisposition to dermatophyte infections, with focus on inheritance in families with a high frequency of dermatophyte infections and specific host–pathogen interactions. The results of studies indicating a hereditary predisposition to dermatophytoses have been challenged by many skeptics suggesting that the varied degree of pathogenicity and the ecological diversity of this group of fungi are more important in increasing sensitivity. Nonetheless, a retrospective analysis of the hereditary propensity to dermatophytoses revealed at least several proven genetic relationships such as races, CARD9 deficiency, HLA-DR4 and HLA-DR8 type and responsible genes encoding interleukin-22, β-defensin 2 and 4 as well as genetic defects in dectin-1, which increased the prevalence of the disease in families and were involved in the inheritance of the proneness in their members. In future, the Human Genome Diversity Project can contribute to elucidation of the genetic predisposition to dermatophytoses and provide more information.

## Introduction

Dermatophytosis is a widespread disease with infection rates reaching 20–25% of the human population each year [1–3]. The major etiological agents of this disease are filamentous fungi called dermatophytes, which have high affinity to keratinized structures, e.g., skin, hair, and nails [1, 4–6]. For many years, the causative agents of dermatophytosis were classified in three genera in the order Onygenales, i.e., *Trichophyton, Microsporum*, and *Epidermophyton* [7]. However, molecular phylogenetic approaches have revolutionized the taxonomy of dermatophytes, demonstrating that *Trichophyton* is a polyphyletic taxon and supporting the introduction of additional genera: *Nannizzia, Arthroderma, Paraphyton*, *Lophophyton, Ctenomyces,* and *Guarromyces* [8].

A dramatic increase in the incidence of dermatophytosis has been observed worldwide over the past two decades. The causes of the increase include such factors as socioeconomic problems, large‐scale international travel, immigration from tropical countries, climate change, and frequent contact with animals, particularly pets [9–12]. In turn, the rise in dermatophytosis-induced morbidity in humans is a consequence of longer life and the inevitable use of immunosuppressive drugs by many patients [13–15]. Moreover, the majority of dermatophytes are zoonotic in nature; hence, close contact with pets generates an increased risk of infection. Therefore, it seems that only an interdisciplinary approach involving dermatologists, pediatricians, primary healthcare physicians, mycologists, and veterinarians can help to curtail the spread of dermatophytoses nowadays [16]. An interesting issue is the role of genetic factors in the predisposition to dermatophytosis and the potential inheritance of these tendencies.

In this review, we describe recent findings about the mechanism of dermatophyte infections, focusing on the genetic predisposition to the disease in humans. The role of inheritance in families with a high frequency of dermatophyte infections and the specific host–pathogen interactions are particularly highlighted.

## Genetic Predisposition among Determinants of Dermatophytosis Prevalence

**Fig. 1:**
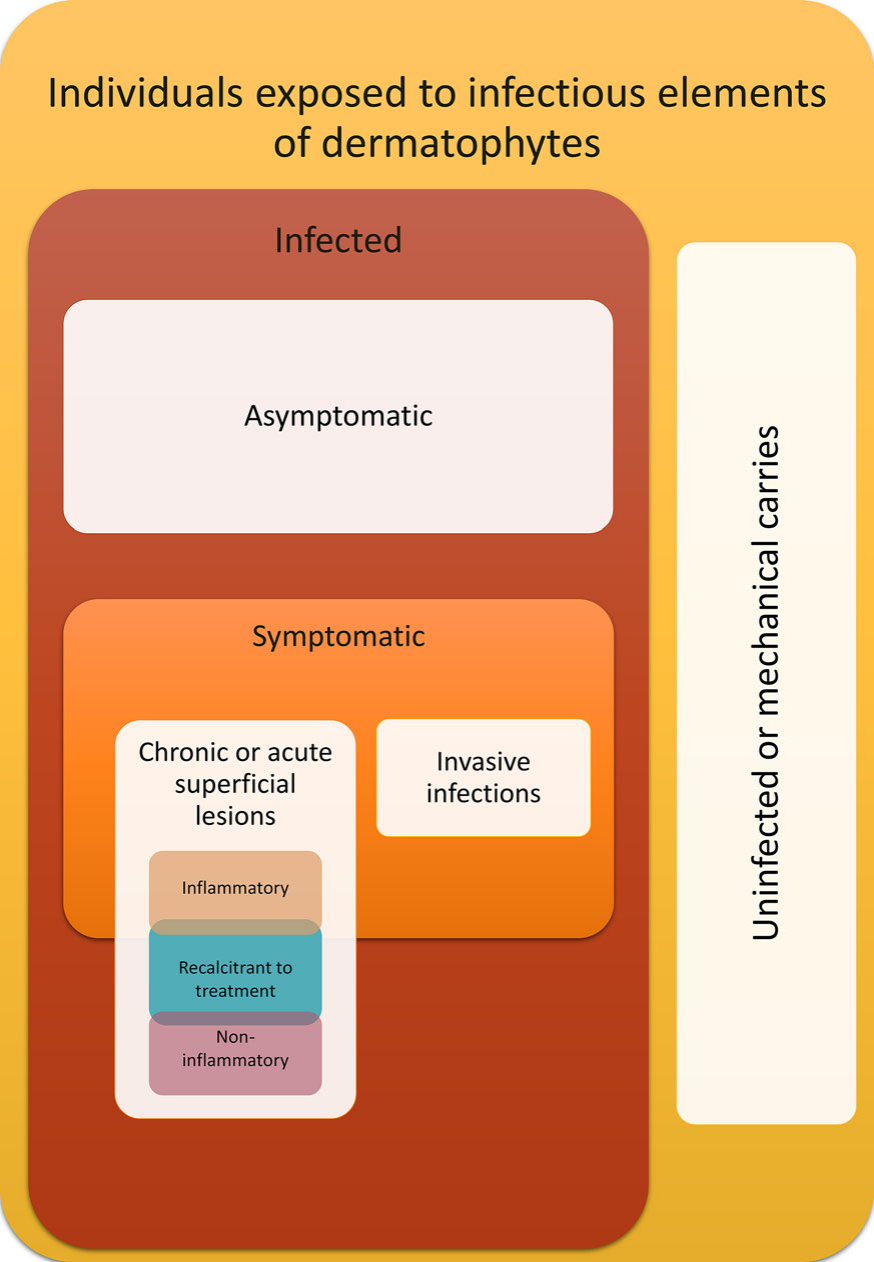
Predisposing factors for dermatophyte infections and their relationships. *Note*: The acquisition of infectious fungal elements does not guarantee infection. Short-term mechanical carriage of the infectious elements of dermatophytes on the skin may pose a risk of transmission. Chronic carriers (months to years) or asymptomatic infections are at higher risk of transmission. An active or symptomatic infection can take two forms: superficial or invasive. The most common superficial infections with two types: inflammatory and non-inflammatory, are described. A special group in both types comprises infections that are recalcitrant to treatment, as reported in recent years

Interestingly, evidence from numerous observational studies indicates that dermatophytes infect humans of every age, race, sex, and socioeconomic status with strikingly high rates [17–19]. Nonetheless, the prevalence of superficial fungal infections is highly variable. The determinants of dermatophyte infection susceptibility and frequency can be divided into three groups, i.e., (1) independent of both the host and the pathogen (environmental), (2) resulting from host’s characteristics, and (3) referring to the species and ecological group of the dermatophyte (Fig. 1) [1, 10, 20, 21]. The first group includes climatic conditions, i.e., humidity and temperature [1, 5, 20–23]. The genetic proneness to infectious diseases together with other factors, e.g., age, sex, maceration of the epidermis, mechanical skin lesions, impairment of immunological barriers, and possible interactions with dermatophytes and their spores associated with the socioeconomic status and profession, is largely dependent on the host [18, 23–28]. Finally, a significant impact on the course of infection is also exerted by the ecological niche occupied by the fungus [23, 24, 29–31].

**Fig. 2:**
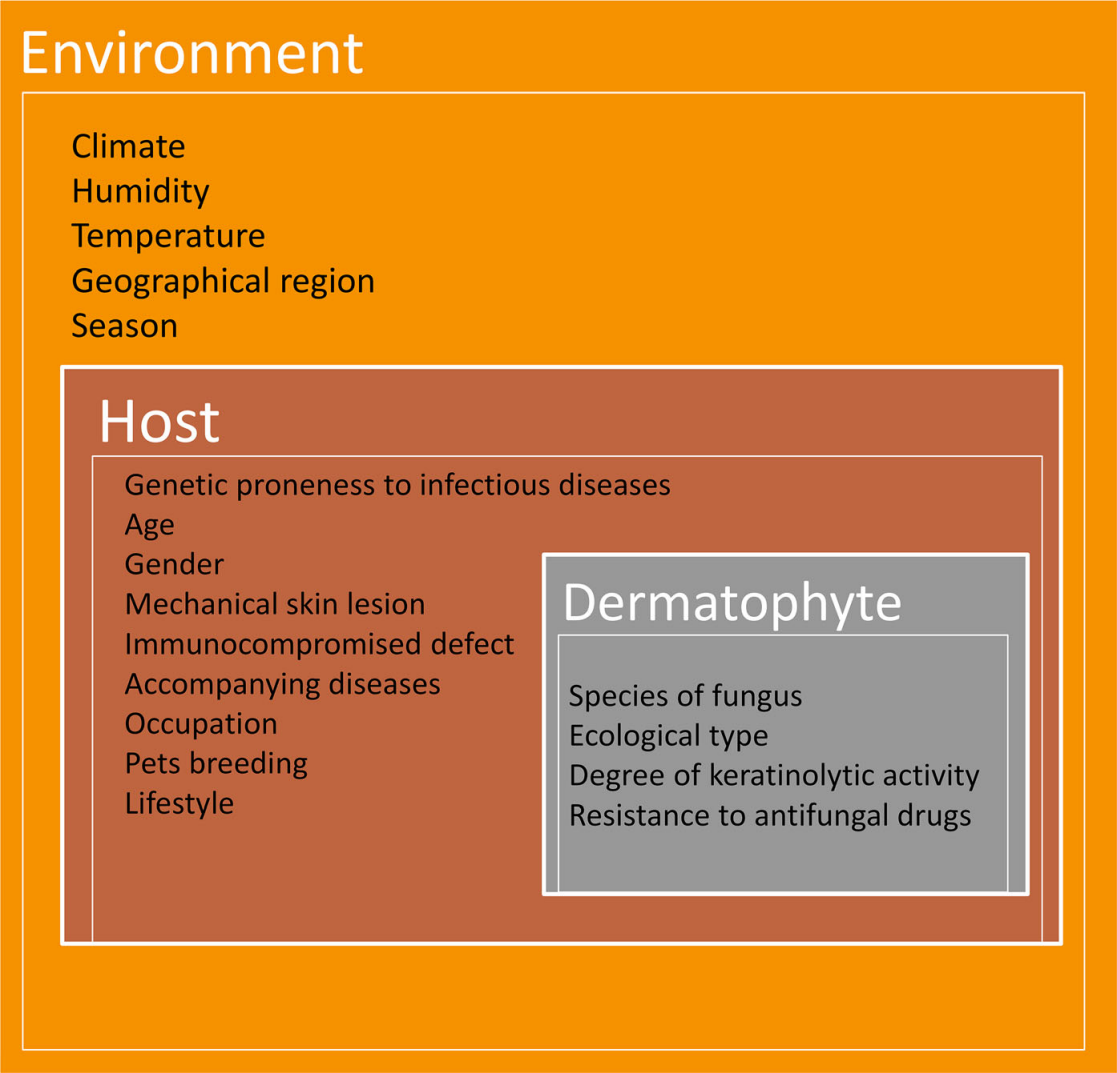
Spectrum of possible effects of the host's interaction with the infectious elements of dermatophytes

The symptoms of dermatophyte infection are not limited to chronic or acute superficial lesions but may represent a wide spectrum from exposed individuals who never develop the infection to those with symptoms that can be inflammatory, non-inflammatory, or recalcitrant to treatment to cases of invasive, disseminated, and life-threatening disease (Fig. 2) [14, 17, 32–38]. Conclusive evidence from observational studies has indicated that the acquisition of dermatophyte infectious elements by the stratum corneum of the host is not synonymous with the occurrence of symptoms of infection [10, 39, 40]. Furthermore, the population of vulnerable hosts is very large and, despite the pathogenic nature of the dermatophytes themselves, the prevalence of cutaneous fungal infections is not as high as might be expected [1, 2, 23, 41, 42]. Secondly, the relatively high prevalence of dermatophytoses in some populations or families may be an important factor proving genetic susceptibility to these fungal infections. In this context, an obvious question is raised: which genetic factors in the human/animal host make some individuals unsusceptible to the development of disease symptoms and even allow them to remain asymptomatic carriers, while others develop severe dermatophytosis that, at the worst, could be invasive or recalcitrant to treatment.

## Host’s Genetic Predisposition or Pathogen Virulence

For almost seven decades, the pathogenesis of infectious diseases has been explored to answer the question whether pedigrees, polymorphism, and other genetic changes, especially the genetics of immunity, underlie the susceptibility to dermatophytes or confer protection against theses pathogens [10, 17, 19]. Although the genetics of the host influences the nature and severity of dermatophyte infections, their relapses despite treatment, and the receptivity of the host physicochemical barrier to the pathogen [43], the pathogenic potential of highly ecologically diversified dermatophytes is equally important in the development of the disease [37, 44, 45]. The highest number of dermatophytosis cases are connected with the presence of animals in the household, and their incidence increases with the growing popularity of pets worldwide [9, 11, 26, 46]. In turn, dermatophytes present in soil have relatively low virulence in comparison with the animal-related pathogens [47, 48]; hence, geophilic dermatophytes are regarded as opportunistic pathogens by many researchers and clinicians [49, 50]. In the center of these pathogenic relations, there are anthropophilic dermatophytes that have adapted to living on keratinophilic substances of human origin, and their high transmission is limited to humans only [10, 51, 52]. However, even within the same ecological group, considerable genomic and phenotypic diversity can be observed among dermatophyte strains, which results from the profile of secreted exoenzymes modulating host response [5, 29, 37, 44, 53, 54]. In addition, due to these differences in the enzyme profile and activity, only some or none of them can be sufficiently virulent to cause symptomatic disease [5, 55–57].

**Fig. 3:**
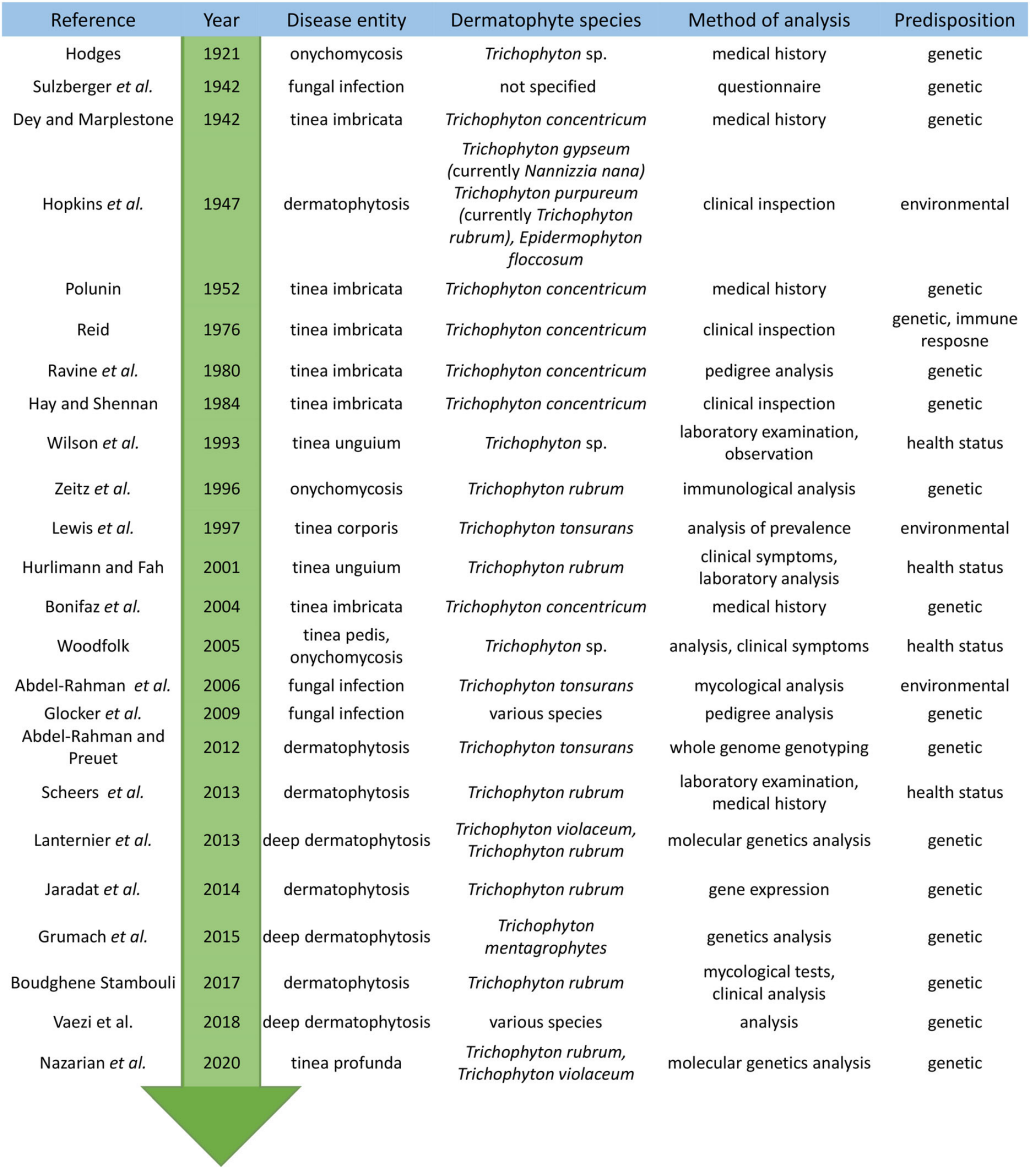
Groundbreaking research about the genetic basis of susceptibility to dermatophytosis

Interestingly, the same genetic dermatophyte strain may exhibit different infection patterns [26, 29, 35, 46, 58]. Additionally, in many cases, different clinical pictures of dermatophytosis have been found in cognate humans living or working together [41, 59, 60]. The host seems to influence the character and extent of the relationship established with dermatophytes upon exposure. An important point in the analysis of genetic predispositions to dermatophyte infections is the selection of a research methodology (Fig. 3). Initially, the genetic basis of susceptibility to dermatophytes was inferred from studies of differences in the incidence of symptomatic infection between genetically related family members and spouses [61–64]. However, these studies were regarded controversial by many investigators, who argued that the differences observed based on medical history and mycological examination could not be relevant. In particular, studies in family members are not reliable due to the high frequency of daily routine contact in the common living environment, which influences the infection rates. A similar prevalence of dermatophytoses is observed in humans who are not familially related but have a common working environment or life activities, e.g., in the army, school, hospital, etc. [41, 42, 64–70].

In their groundbreaking study, Abdel-Rahman et al*.* [58] have suggested that the cross-sectional sampling strategies favored in most epidemiologic studies are inadequate for describing the natural course of infection and fail to identify individuals that develop active disease; therefore, a different strategy to test the predisposition to infection should be implemented. The authors evaluated preschool-aged children attending the same child care center in a 2-year prospective longitudinal study [58]. Then, molecular strain typing was conducted to discriminate clearly between individuals that had never acquired the pathogen, those that had intermittently acquired and lost different fungal strain types, and those that had acquired and sustained infection with the same strain type for years. In their study, 3541 scalp cultures were collected from 446 children. Twenty-two percent to 51% of the scalp cultures per month were positive, yielding 1390 fungal cultures with 1048 of typeable ones. Among children with multiple typeable isolates, 51% exclusively carried the same strain, 37% had a single predominant strain with secondary strains acquired transiently, and 12% harbored a different strain of *T. tonsurans* in each typeable culture. The probability that the same strain persisted in subsequent months was almost 90%, which was unlikely to have arisen by chance. The rates of symptomatic disease were significantly different between the exclusive, predominant, and transient carriers of *T. tonsurans.* In contrast to dermatophyte infections in older individuals, where symptomatic disease seems to be a consequence of pathogen acquisition and asymptomatic carriers can be traced to the index case, the infection in the examined preschool-age population was endemic, and symptomatic disease seemed to have been activated by a single strain that persisted on the scalp.

Indeed, it has been acknowledged that the health condition of the host influences the nature and extent of the relationship established with dermatophytes, and the same genetic dermatophyte strain can induce different infection patterns in humans sharing the same environment [2, 41, 58]. In addition, some accompanying health conditions such as eczema, psoriasis, ichthyosis, atopic dermatitis, and seborrheic dermatitis may influence the susceptibility to dermatophytes [58, 71–76]. Therefore, consideration of the genetic basis of susceptibility to dermatophyte infections without assessment of the pathogenicity of the fungus itself does not seem to have solid grounds. Finally, it is possible that different strains of fungi with different infective capacities are responsible, which in combination with the genetic susceptibility of the host organism determines the type of infection caused. Thus, the host's genetic predisposition is as important as the degree of pathogen virulence and adaptation. In clinical practice, both factors should be taken into account and mycological tests should always be performed correlating their results with the patient's condition.

## Inheritance of Susceptibility to Dermatophyte Infection

Regardless of the methodological errors described above, the research on the prevalence of dermatophyte infections in related humans provides grounds for discussion. Contradictory conclusions on hereditary trends in dermatophytoses were formulated in investigations conducted in the middle of the twentieth century. The studies showed differences in the rates of dermatophyte infections between genetically related family members, husbands/wives marrying into these families, and completely unrelated people living in the same environment [62, 63, 77–79]. Sulzberger et al*.* [80] assessed the extent of familial infections of feet and groins on the basis of questionnaires completed by over 100 dermatologists. Their study revealed a very low relationship between family-related members and the prevalence of dermatophytoses, with only four proved cases of familial infection among hundreds of thousands of patients examined; therefore, such a frequency of infection had no practical importance. The authors declared that no familial infection was proved unless the fungi were isolated in the culture and shown to be morphologically similar. These associative studies in family members were confounded by the frequent contact in a shared environment, which has been shown to have an impact on infection rates in populations without genetic relationships [66, 67, 78, 81]. The prevalence of dermatophytoses in people living or working together has become a criterion in further research of the genetic determinants of predisposition to these diseases conducted by skeptics. In their investigations of fungal infections of the feet of soldiers at a military post, Hopkins and Hillegas [78] found that various species occurred at approximately the same ratio in most of the groups examined. Therefore, individual susceptibility to an existing latent infection and immunity were more important than the risk of cross-infection. Nonetheless, doubts about the accuracy of the conclusions were aroused by the fact that three of the 26 groups analyzed in their study showed considerable variation in the predominant fungal species causing the infection, while others showed only slight variation. The discrepancies in these results were related to the rapid turnover of personnel in military establishments, which often did not ensure sufficient time for any species to become dominant [78]. Moreover, the prevalence of fungal skin infections is also high in contact sports, with tinea gladiatorum in wrestlers as a frequently cited example [70, 82]. However, a higher rate of dermatophytosis was observed in athletes under 15 years old [82, 83]. Probably, the high levels of the fungistatic action of steroid hormones occurring after puberty may reduce the incidence of tinea gladiatorum in the older age range despite continuation of the wrestling activity [83]. Lewis and Lewis [64] identified healthcare workers in a rehabilitation hospital with identifiable physical contact, and estimated the incidence of the fungal infection at 25% in this group. The infection rate was 33% in the major contact group and 17% in the moderate contact group; no infections were noted in the minimal contact group.

However, these historical observations with analysis of more advanced pedigree results provide some evidence for the genetic association of susceptibility to dermatophyte infections. The first important conclusion was formulated by Bonifaz et al*.* [84]. In their study on confirmed cases of *tinea imbricata* caused by *Trichophyton concentricum*, the genetic susceptibility to dermatophytosis was found to represent autosomal dominant inheritance in nine out of 16 family members, i.e., children that had the same mother but different fathers. Although autosomal recessive inheritance of the susceptibility to *tinea imbricata* was reported already in 1980 by Ravine et al*.* [85], the authors definitely argue that the presented family case strongly indicates an autosomal dominant rather than recessive inheritance pattern of susceptibility. Furthermore, these contradictory conclusions were not fully comprehensive to clarify the genetic predisposition to *tinea imbricata.* As indicated by Hay et al*.* [86], except the inheritance of susceptibility to this disease, also heritable ineffective immune response to the infection may be an explanation of the high relapse rate and the extensive character of clinical lesions occurring in *Tinea imbricata*. Dey and Maplestone [87], Polunin [88], and Reid [89] reported a much higher prevalence of *tinea imbricata* in some races living in the same country and in closely related environmental conditions and highlighted the presence of racial characteristics in the susceptibility to the disease. The observations of the racial dependences in the infection rate may be a result of ethnically induced environmental differences between the racial groups of people living in close proximity to each other.

Moreover, in their study of the pedigrees of families in Italy with distal subungual onychomycosis and concomitant *tinea pedis* caused in foot soles by *T. rubrum*, Zaias et al*.* [90] found that the autosomal dominant pattern of inheritance was crucial in increased sensitivity. Additionally, the frequency of infections in family members with hereditary tendencies was equal in both sexes. Similar observations of the heredity of onychomycosis tendencies were made in families residing in France. Onychomycosis is interesting from one more point regarding genetic propensities; namely, in almost 50% of cases diagnosed in children, parents were also affected, which suggests genetic predispositions [19].

In many studies, a breakdown in the immune response was also proposed as the cause of the differences in the susceptibility to dermatophytes. The differential diagnosis for severe, deep, recurrent cutaneous fungal infections in patients that are non-immunosuppressed or do not receive immunosuppressive treatment includes especially CARD9 (caspase recruitment domain-containing protein 9) deficiency due to compound heterozygous mutations [91–93]. CARD9 is an adaptor molecule that drives the antifungal activity of macrophages and neutrophils in the skin [91]. Additionally, this deficiency is inherited in an autosomal recessive manner [94]. Moreover, CARD9 is indispensable for the activity of T-helper 17 (Th17) cells, mostly through dectin-2, to a lesser extent dectin-1 signaling, macrophage-inducible C-type lectin, and probably other yet unknown receptors involved in immunity [95–97]. Glocker et al*.* [98] showed a pedigree analysis for a consanguineous family with multiple members affected by chronic fungal infections associated with a presumably CARD9 autosomal recessive mode of inheritance. In their study, recurrent fungal infections were diagnosed clinically in eight family members, three of whom died in early adolescence. None of these patients had unusual bacterial or viral diseases, which proves that the host defense against these pathogens was normal. Furthermore, investigations conducted by Nazarian et al*.* [93] revealed a *tinea profunda* case caused by *Trichophyton rubrum* and *Trichophyton violaceum* in a 31-year-old-man associated with bi‐allelic mutations in CARD9. Vaezi et al*.* [96] reported that deep dermatophytosis accounted for 37.3% of all reported cases of fungal infections linked to CARD9 deficiency due to autosomal recessive mutations. *Trichophyton violaceum*, *T. rubrum*, and *T. mentagrophytes* were observed as etiological agents of these dermatophytoses [93, 99–101]. Interestingly, analyses of the characteristics, distribution, frequency, and relationship between the genotype of the CARD9 gene mutations and fungal infections in the reported cases revealed that dermatophytosis related to this factor encompassed up to 75% of African cases [96], which are likely reflected in the high prevalence of *T. violaceum* isolation on this continent [48, 102]. These data suggest that mutations may be specific in some populations or geographic regions where the high rate of consanguinity has been noted in many closed groups.

In geographically distant populations of patients, the adaptive immune response to dermatophytosis has been widely studied [75, 103–107]. The major histocompatibility complex (MHC) and the HLA (Human Leukocyte Antigen) system are considered critical for the presentation of antigens and activation of T cell-mediated responses in the course of fungal infection [75]. In a Brazilian Ashkenazi Jewish population with *T. rubrum* onychomycosis, Zaitz et al*.* [103] observed that HLA-DR4 was found in 100% of individuals without symptoms and in 25% of cases, thus implying a protective effect against the susceptibility to the disease. In turn, in a Mexican mestizo population with onychomycosis caused by the same dermatophyte species, García-Romero et al*.* [104] determined a higher frequency of HLA-DR8 in the families with the disease, suggesting that this haplotype might confer the susceptibility. Furthermore, Carrillo-Meléndrez et al*.* [75] showed association of HLA-DR8 with the genetic susceptibility to development of onychomycosis in nail psoriasis patients. Their study also revealed possible association of HLA-DR1 with the genetic predisposition to development of onychomycosis.

This retrospective analysis of the hereditary determinants of the predisposition to dermatophytoses indicates that the prevalence of some disease entities associated with this group of fungi is substantially higher when the genetic element is involved. Moreover, the geographic aspect of these relationships closely related to human races living in close proximity to each other is emerging. In addition, identification of high-risk families will allow education of their members about the risk of fungal infections. In turn, in clinical practice, this information given at the interview can shorten the diagnosis and prompt the dermatologist to apply an appropriate therapy. On the other hand, an overly general approach considering dermatophytosis as a whole and the use of methods based solely on infection frequency analysis is unreliable and leads to erroneous conclusions on the genetic predisposition.

## Identification of Genes Involved in Susceptibility

The statement that the genetic susceptibility to dermatophytosis is a monogenic feature can be as erroneous as the trend to insist on the important role of genetics factors and disregarding other host, pathogen, or environmental influences. One of the main limitations of studies of the genetic predictors of susceptibility to anthropophilic dermatophytes is the search for genetic dependencies ignoring non-inherited factors [17]. Thus, the search for genes responsible for increased susceptibility to dermatophytosis is challenging, as the pathogen–host interaction should be treated holistically [10, 28].

Interestingly, successful tactics for detection of genes responsible for host–pathogen interactions and thus associated with increased susceptibility to dermatophyte infections was undertaken by Abdel-Rahman and Preuett [108]. In their study, extensive search for genes that may be linked with infection was conducted as part of a genome-wide association study in a cohort of children in whom the frequency of *tinea capitis* infection was characterized longitudinally over several years. The study involved 20 children who carried *T. tonsurans* > 90% of the time and 20 children who carried the fungus < 10% of the time. Generally, the authors identified 21 genes with a genotype associated with carriage of the fungus, although they did not study whether this was correlated with symptoms of *tinea capitis*. The genes uncovered in this study were associated with various different functions, including leukocyte function, remodeling of the extracellular matrix, wound repair, and cutaneous permeability (Table 1). The risk index assigned to the genotypes in these 21 genes accounted for over 60% of the variability observed in the infection rate, and eight of all the analyzed genes appeared to account for the majority of the observed variability in the susceptibility to dermatophyte infections [108].

**Table 1:** Host genes with variability degree of expression noted during dermatophyte infection

Functions	Genes	References
Extracellular matrix formation, integrity, and remodeling	FBLN5	Abdel-Rahman and Preuett [108] (104)
	FBN2	
	MFAP4	
	SMOC2	
	PCDH7	
	MMP3	
	ADAM12	
Recruitment, activation, and migration of leukocytes	SEMA6A	Abdel-Rahman and Preuett [108] (104)
	ROBO1	
	SLIT3	
	cd99L2	
	CSMD1	
	GAB2	
Epidermal development, maintenance, and wound repair	FGF1	Abdel-Rahman and Preuett [108] (104)
	MAPK8	
	IGF1R	
Skin homeostasis and interaction with pathogen	LASS4	Abdel-Rahman and Preuett [108] (104)
	GALP	
	KAL1	
	FibCD1	
Involved in innate immune response	hBD-2	Jaradat et al. [109] (105)
	DEFB4	
	Interleukin-22 gene	
Recognition of fungal β-glucan by the receptor dectin-1	CLEC7A-Y238X	Ferwerda et al. [113] (109)

These findings indicate that genetically determined deficiency in adaptive immune responses may affect the predisposition to dermatophyte infections. In addition, literature reports have revealed possible interactions in the prevalence, i.e., a defect in the innate response may impair the adaptive response, thus potentiating the susceptibility [19, 43, 109, 110]. Jaradat et al*.* [109] investigated the association of *T. rubrum* dermatophytosis with the expression of genes that encode IL-22, human β-defensin 2 (hBD-2), and β4-defensin (DEFB4) (Table 1). Their findings indicated an association between the variation in the number of copies of DEFB4 mRNA and the occurrence of *T. rubrum*–induced superficial dermatophytosis. The authors hypothesized that a low DEFB4 copy number was a risk factor for dermatophytosis, together with elevated IL-22 levels implicated in its pathogenesis. Other studies revealed dermatophytosis candidate genes other than DEFB4, such as the Fc receptor gamma, which is used by the pattern recognition receptor dectin-2 to induce innate immune responses against *T. rubrum* [111, 112]. This gene has also shown a variable number of copies in humans with or without infections and may similarly contribute to the pathogenesis of dermatophyte infection.

Other studies of a candidate gene for predisposition in patients suffering from superficial dermatophytosis indicated CLEC7A-Y238X, i.e., an early stop codon variant that influences the recognition of fungal β-glucan by the receptor dectin-1 [113]. The defective surface expression of dectin-1 related to the Tyr238X polymorphism resulted in failure of β-glucan recognition and an impaired cytokine response by monocytes and macrophages [114]. The identification of this polymorphism in all African populations assessed suggests that this is an ancient mutation that most likely emerged more than 60,000 years ago, before the split of the modern human populations in the late Paleolithic [115]. In Europe, such a genetic defect was reported in a Dutch family where all members were affected by onychomycosis [113].

The Human Genome Diversity Project (HGDP) can contribute to elucidation of the genetic predisposition to dermatophytoses and provide further information about the frequency and world distribution of the genomic polymorphism in connection with prevalence of this infectious disease [116]. Such analyses of the haplotype diversity within the white population and other races, families, and populations by studying genome-wide polymorphism datasets will certainly reveal other genes that are keys to increased sensitivity [43].

## Conclusion

The heterogeneous nature of dermatophytes and their hosts indicate that the susceptibility to infection is probably a cumulative result of changes on both sides and their mutual adaptation. Moreover, multiple studies have revealed a role for host genetics in the development of illness, including possible Mendelian inheritance patterns for predisposition of dermatophytosis. In our review, the complexity of the genetic interaction between dermatophytes and their natural and incidental hosts is highlighted. Literature analysis shows that different models and methodologies may lead to divergent interpretations of this relationship. The choice of an appropriate model for analysis and inferences is a critical step in understanding these pathogens better. There is also a wide field for examining the genetic of immune response of the host to the dermatophyte infection. Future studies will require broader exploration of the dermatophyte genome in combination with analysis of large phenotypically well-characterized populations of various dermatophyte species to identify the main factors mediating the infection risk that can be directed to disrupt host–pathogen interactions and used in therapies. Therefore, extensive studies on the interactions between dermatophytes and their specific hosts, which involve complex molecular mechanisms, have high theoretical and application significance. However, it seems indisputable that genetic predisposition plays an important role in the susceptibility to dermatophyte infections.
